# The mechanisms of wine phenolic compounds for preclinical anticancer therapeutics

**DOI:** 10.29219/fnr.v65.6507

**Published:** 2021-08-23

**Authors:** Jing Duan, Hua Guo, Yulin Fang, Guangbiao Zhou

**Affiliations:** 1 College of Enology, Northwest A&F University, Yangling, China; 2 State Key Laboratory of Molecular Oncology, National Cancer Center/National Clinical Research Center for Cancer/Cancer Hospital, Chinese Academy of Medical Sciences and Peking Union Medical College, Beijing, China

**Keywords:** wine, cancer, phenolic compounds, mechanism, chemotherapy

## Abstract

**Background:**

Wine is one of the oldest and most popular drinks worldwide, which is rich in phenolic compounds. Epidemiological studies show that moderate consumption of wine can reduce the risk of certain diseases, and this effect is attributed to its phenolic compounds.

**Objective:**

The objective of this review was to elaborate the effects of wine-derived phenolic compounds for preclinical anticancer therapeutics and their major mechanisms.

**Methods:**

In this review, we discuss the classification and content of common phenolic compounds in wine and summarize previous studies that have evaluated the anticancer properties of wine-derived phenolic compounds and their mechanisms.

**Results:**

Wine-derived phenolic compounds have been proven to participate in several mechanisms against cancers, including deoxyribonucleic acid damage, oxidative stress, cell proliferation, cell cycle arrest, cell apoptosis, autophagy, cell invasion and metastasis, immunity and metabolism, regulation of multiple signaling molecules, and gene expression. However, the exact anticancer mechanisms of the phenolic compounds in wine need to be further investigated.

**Conclusion:**

Wine-derived phenolic compounds are promising chemoprotective and chemotherapeutic agents for cancer.

## Popular scientific summary

- Wine phenolic compounds have an important impact on human health.
- Wine phenolic compounds may act against cancers via modulating multiple biological mechanisms.
- The relationship between wine consumption and health still remains controversy.

**Fig. 1:**
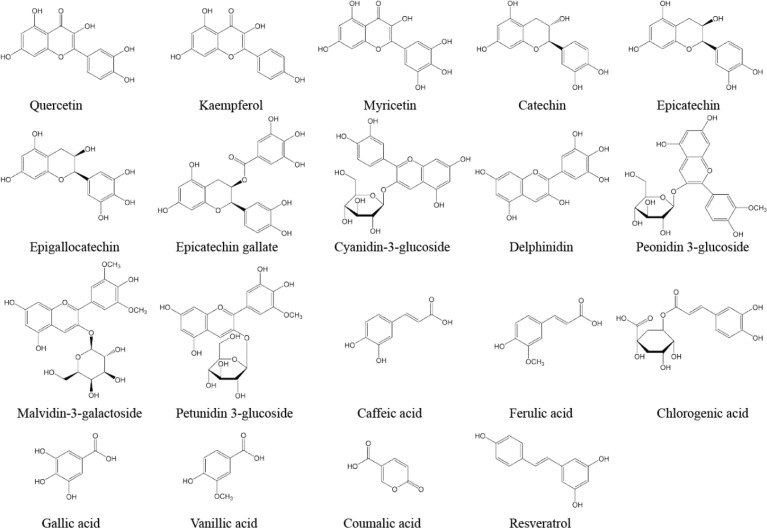
Chemical structures of the phenolic compounds frequently found in wine.

Wine is an alcoholic beverage obtained from the fermentation of the juice of freshly gathered grapes. The composition of wine is significantly different from that of grape juice. One of the reasons could be ascribed to the fact that some components of grapes are discarded during winemaking. Another reason could be attributed to the complicated series of transformations and processes related to winemaking, which affect the final product. Red wine, especially, is a rich source of phenols and represents an important dietary ingredient for human consumption (1). The main phenolic compounds found in wine are flavonoids and non-flavonoids. The most common flavonoids in red wine are flavonols, flavanols, and anthocyanins, while the commonly occurring non-flavonoids are mainly derivatives of hydroxycinnamic and hydroxybenzoic acids, hydrolyzable tannins, and stilbene (2), as shown in Table 1. The chemical structures of the phenolic compounds frequently found in wine are shown in Fig. 1.

**Table 1:** The main phenolic compounds in red wine

Basic type	Concentration	Common component	Reference
Flavonols	50–200 mg/L	Quercetin	(3, 4)
		Kaempferol	
		Myricetin	
Flavanols	40–120 mg/L	Catechin	(3, 5, 6)
		Epicatechin	
		Epigallocatechin	
		Epicatechin gallate	
	500–1,500 mg/L	Proanthocyanidins	
Anthocyanins	90–400 mg/L	Cyanidin	(3, 7)
		Delphinidin	
		Peonidin	
		Malvidin	
		Petunidin	
Hydroxycinnamic acids and hydroxybenzoic acids	60–240 mg/L	Caffeic acid	(3, 8)
		Ferulic acid	
		Chlorogenic acid	
		Gallic acid	
		Vanillic acid	
		Coumalic acid	
Hydrolyzable tannins	0–260 mg/L	Ellagitannins	(3, 9)
		Gallotannins	
Stilbene	0–7 mg/L	Resveratrol	(10)

Currently, the treatment modalities for cancer include surgery, immunotherapy, radiation therapy, targeted therapy, and chemotherapy. The two most important factors considered during chemotherapy are the dose and toxicity of the drug. Many naturally occurring phenols are efficacious at low doses and are not associated with significant toxicities. Epidemiological and clinical studies show that moderate wine consumption has chemopreventive effects and also exhibits therapeutic effects in individuals with cardiovascular disease, hypertension, diabetes, and cancers (1). In 1997, Jang et al. (11) confirmed that phenols are the functional substances in wine. Since then, numerous studies have shown that the positive effects of wine on health are attributed to its polyphenolic compounds, as these compounds exhibit antioxidant, anti-inflammatory, hypotensive, anticoagulant, and even anticancer effects (12–14). The beneficial effects of these compounds in some diseases are related to the ability of phenolic compounds to scavenge reactive oxygen species (ROS), nitrogen radicals, and chlorine species (15, 16). However, the chemopreventions of certain diseases, especially cancers, are challenging as they are related to the interaction of proteins and involve complex cell signaling pathways (17, 18). In recent years, many phenolic compounds in wine have been proven to have prophylactic or therapeutic effects on various types of cancers, including cancers of the colon (19), ovary (20), breast (21), lung (22), and prostate gland (23).

The phenolic compounds in wine have been shown to have anticancer effects, and resveratrol is the most studied compound. The phenolic compounds in wine are known to play a role in inducing cell cycle arrest, apoptosis, autophagy, deoxyribonucleic acid (DNA) damage, and p53 signaling, all of which eventually lead to the death of cancer cells (24). Grape seed extract or red wine polyphenolic compounds can inhibit certain cancer cells by regulating the immune and metabolic systems, such as targeting the enzymes involved in arachidonic acid metabolism in colorectal cancer (25). Several factors alter cell signaling pathways; however, the phenolic compounds in wine, such as flavonoids, can reverse and restore normalcy (18). Currently, more novel mechanisms are needed to prevent and treat cancers. The anticancer effects exhibited by the phenolic components of wine appear complicated as many different pathways are involved and many unidentified mechanisms have yet to be studied.

In this review, we present recent findings pertaining to the anticancer properties of the phenolic compounds of wine. We also summarize protective effects and mechanisms of main bioactive phenolic compounds in wine with a focus on flavonoids and resveratrol.

## Wine consumption and health: controversy remains

**Fig. 2:**
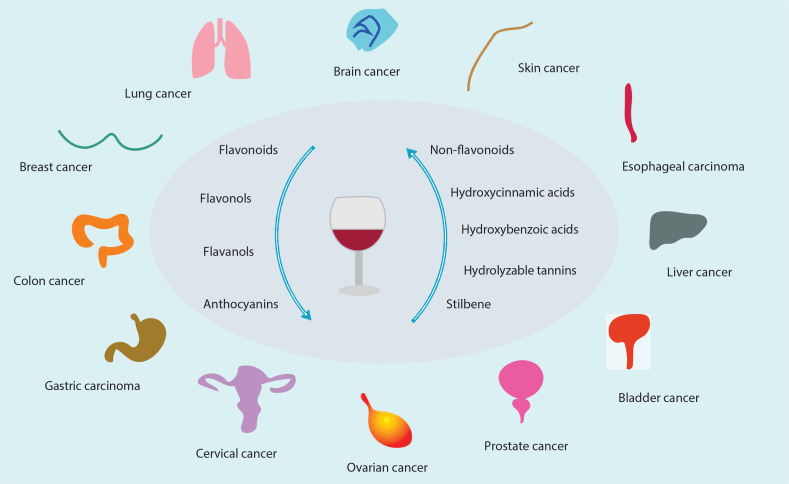
The major phenolic compounds in wine and the types of cancer that phenolic compounds exert anticancer effects.

A 12-year follow-up study showed that low to moderate alcohol consumption, namely 10–14 drinks peer week, was correlated with better total and individual cognitive functions, including word recall, mental status, and vocabulary among 19,887 participants in United States adults (26). Moderate alcohol consumption such as a small glass of wine daily could reduce the risk of ischemic heart disease, diabetes, and ischemic stroke (27, 28). In addition, a systematic review and meta-analysis demonstrated that low to moderate alcohol consumption is associated with a reduced risk of 0.75 folds for cardiovascular disease and coronary heart disease mortality, and 0.71 folds for incident coronary heart disease (29). However, another study was conducted to analyze the relationship between lifestyle and genetic risk with the incidence of dementia in a total of 196,383 participants with a mean age of 64.1 years, and the results showed that both an unfavorable lifestyle like moderate alcohol consumption and high genetic risk are strongly associated with higher dementia risk (30). A translational study indicated that chronically alcohol consumption expands mean diffusivity of brain gray matter among humans and rats and is positively associated with a clear decrease in extracellular space tortuosity caused by a microglial reaction (31). However, the relationship between wine consumption and cancer risk still remains elusive. Alcohol consumption is reported to be able to increased risks of cancer, including cancers of the breast, lip, and oral cavity (32). Similarly, a global systematic analysis study regarding the alcohol use and disease burden demonstrated that alcohol consumption is a leading risk factor of cancers (33). On the contrary, some studies suggest that alcohol intake of reasonable quantities, such as low to moderate consumption of wine, may be beneficial for the treatment of several types of cancer (27, 28). The anticancer properties of the phenolic compounds in wine are shown in Fig. 2.

## The anticancer effects of flavonoids and their potential mechanisms

Flavonoids are characterized by a C_6_-C_3_-C_6_ skeleton, which contains two benzene rings that are connected by a pyran ring in the center. Most phenolic compounds in red wine are flavonoids. The total phenolic content is more than 85% in red wine but less than 20% in white wine (34). Flavonols, flavanols, and anthocyanins are the subclasses of flavonoids present in wine. The consumption of flavonoids may reduce the incidence of cancers and improve prognosis. They can inhibit the progression of several types of cancer through inducing programmed cell death, inhibition of cell growth and viability, cell cycle arrest (35, 36), and interfering with the metabolic functions associated with the aberrant immune function (37).

### Flavonols

Flavonols are a group of yellow phenolic pigments belonging to the flavonoid family that are found mostly as glycosides, such as glucosides and glucuronides. Flavonols have been shown to inhibit tumorigenesis in *in vitro* and *in vivo* models. The most common flavonol in grapes is quercetin, which is present in its skin and stem (38). In the past decades, several anticancer properties of quercetin have been discovered, such as its cell signaling, proapoptotic, antiproliferative, antioxidant, and cell-growth suppression effects. Quercetin exerts its primary antioxidant effect by providing electrons to reduce the levels of ROS, inhibiting tumor cell growth and preventing DNA damage caused by mutations (39). In addition, quercetin exhibits proapoptotic effects in tumor cells through mitochondria-mediated pathways or by increasing cytotoxicity and oxidative stress (40, 41). Quercetin also inhibits the proliferation of human hepatoma cell line, HepG2, by altering the expression of the cytochrome P450 proteins A1 (*CYP1A1*) gene (42). At low concentrations, quercetin inhibits the proliferation of human breast cancer cells by arresting the cell cycle in G1 phase (43). Additionally, quercetin increases G2/M phase and the levels of p53 and p21 proteins and induces cytotoxicity and apoptosis in lung carcinoma cells (44). Several experimental studies indicate that quercetin may be a promising adjuvant in the chemotherapy of cancers. Quercetin promotes cisplatin-induced apoptosis in oral squamous cell carcinoma by reducing the nuclear factor κB (NF-κB) and x-linked inhibitor of apoptosis (xIAP) protein levels and results in the significant inhibition of tumor growth (45). NF-κB is important for the differentiation of immune cells and is involved in metabolic disorders (46). When combined with metformin, quercetin strongly inhibits the growth, migration, and invasion of prostate cancer cells both *in vitro* and *in vivo* through the vascular endothelial growth factor/AKT8 virus oncogene cellular homolog/phosphatidylinositol-3-kinase (VEGF/Akt/PI3K) signaling pathway (47). Furthermore, quercetin can regulate the activity of several tyrosine kinases. In general, the anticancer effects of quercetin are owing to its ability to induce apoptosis, arrest the cell cycle, scavenge free radicals, and regulate proteins such as tyrosine kinases, p53, and heat shock proteins (HSPs) (48). Moreover, it is reported that quercetin modulates immunity to promote the anticancer response by regulating dendritic cells (DCs) activation and decreasing tumor necrosis factor (TNF), interleukin-1β (IL-1β), IL-6, IL-10, and IL-12p70 secretion (49). Poor water solubility and low bioavailability of quercetin are the limiting factors of its application in a clinical setting for cancer chemoprevention (50). However, quercetin is an indispensable food ingredient, and its bioavailability increases when derived from sources, such as onions or grapes. In the future, nanoparticles or other modes of drug transport will be essential for the effective delivery of quercetin to cancer cells (51).

Besides quercetin, the common flavonols in wine include kaempferol and myricetin. The structures of these compounds are similar. Myricetin, also known as hydroxyquercetin, has an additional −OH group compared to kaempferol. Epidemiological studies show that kaempferol and myricetin exhibit therapeutic potentials in different types of cancers, including cancers of the bladder, stomach, colon, ovary, pancreas, lungs, breast, and the prostate gland (52–54). They inhibit growth, migration, and invasion of cancer cells and induce apoptosis by activating or inhibiting different signaling pathways and reactivating various key genes involved in tumorigenesis. In breast cancer, kaempferol arrests the cell cycle in the G2/M phase by decreasing the level of cyclin-dependent kinase 1 (CDK1) (55) and inhibiting migration and invasion by blocking the PKCδ (protein kinase C)/mitogen-activated protein kinase (MAPK)/activator protein 1 (AP-1) cascade and the expression of MMP-9 (matrix metalloproteinases) (56). Kaempferol induces apoptosis by regulating caspase-3 expression and the cleavage of poly-ADP-ribose polymerase (PARP), B-cell lymphoma-2 (BCL-2), and Bax (57). Other findings reveal that ROS are the reason for kaempferol in inducing apoptosis (58). Moreover, recent studies demonstrate that kaempferol induces autophagy and cell death by increasing the conversion of light chain 3 (LC3)-I to LC3-II and decreasing the expression of p62 in gastric, hepatic, and lung cancers (59–61). Kaempferol activates the inositol-requiring enzyme 1 (IRE1)1/Jun n-terminal kinase (JNK)/C/EBP homologous protein 10 (CHOP) pathway from the cytosol to the nucleus and promotes autophagic cell death by inhibiting the histone deacetylase (HDAC)/G9a axis in gastric cancer (62). In hepatic cancer cells, kaempferol induces autophagy through the AMP-activated protein kinase (AMPK)/AKT pathway and decreases the expression of CDK1/cyclin B (61). Myricetin may target a specific molecule or multiple signaling pathways to inhibit tumor progression. It is reported that myricetin suppresses breast cancer metastasis by downregulating MMP-2/9 and mRNA ST6GALNAC5 levels *in vitro* and *in vivo* (63). Myricetin induces apoptosis in ovarian, skin, and colon cancer cells by upregulating the proapoptosis proteins including Bax, BCL-2, cleaved PARP, and caspase-3 (54). In addition, myricetin was found to downregulate extracellular signal-regulated kinase (ERK)/p90RSK/AP-1, janus-family tyrosine kinase 1 (JAK1)/signal transducer and activator of transcription (STAT), and PI3K/AKT pathways in cancer cells (64). Myricetin demonstrates the immunomodulatory effects by inhibiting T cell proliferation and reducing the secretion of interferon-γ (IFN-γ), IL-2, and IL-17 (65). However, the immunomodulatory effects of cancer immunity by myricetin *in vivo* are yet to be studied.

### Flavanols

Flavanols, a subclass of flavonoids, are hydroxylated at C3 in the heterocyclic ring. Flavanols are often referred to flavan-3-ols and comprise catechin, epicatechin, epigallocatechin, epicatechin gallate, and proanthocyanidins, which are present in red wine. They react with tannins and lend wines their characteristic flavor (66). Studies suggest that flavanols can reduce the risk of various diseases by maintaining glucose homeostasis, exerting a prebiotic effect on gut microbiota, and enhancing insulin signaling (67). The inhibitory effects of catechin against cancers have been demonstrated in many studies, and prostate cancer is one of the most studied cancer types in anticancer research involving catechin. Catechin has direct as well as indirect effects on cancer cells, and the latter involves affecting the tumor microenvironment (68). The main anticancer inhibitory actions of catechins are by inducing apoptosis in different animal models, reducing the phosphorylation of c-Jun and Erk1/2 levels in lung tumorigenesis models, suppressing phospho-Akt and nuclear β-catenin levels in colon cancer models, inhibiting the insulin-like growth factor-1 (IGF1)/IGF1 receptor (IGF1R) axis in colon and prostate cancer models, and restraining the VEGF-dependent angiogenesis in lung and prostate cancer models (69–71).

Epicatechin exerts antiproliferative effects in gastric, prostate, ovary, and lung cancers and is able to reduce cisplatin-induced toxicity (72). Epicatechin has been reported to regulate mitochondrial activity by inhibiting phosphorylated Erk2 associated with Ras/MAPK signaling pathway at low concentrations (73). Thus, epicatechin can induce cancer cell death by increasing cell stress and sensitivity. Mitochondrial respiration, ROS production, and Warburg metabolism are the likely mechanisms of action. In addition, epicatechin was found to inhibit the expression of NF-κB, AP-1, Akt, and nuclear factor erythroid-2 related factor 2 (Nrf2) pathways, which are important in cell proliferation and survival (74). Mice were treated with either vehicle control group, 1 mg/kg epicatechin group, or 5 mg/kg naltrindole (a δ-opioid receptor antagonist) for 10 days, and the results showed that mice of epicatechin-treated group had the highest respiration rates, suggesting that epicatechin has the potential to augment mitochondrial function (75). Epigallocatechin and epicatechin gallate, which have structural similarities with epicatechin, also showed inhibitory effects in oral and prostate cancer through different signaling pathways (76). Epigallocatechin gallate has been reported to inhibit cancer by modulating immune by decreasing Th1 and CD8^+^ T cells and increasing Tregs (77).

Proanthocyanidins comprise a group of oligomers and polymers of flavanols in grape seeds, which have potent antioxidant and antitumorigenic properties owing to their ability to target multiple oncogenic signaling pathways (78). Epidemiological and clinical studies show an inverse association between proanthocyanidin intake and prostate cancer risk in 43,000 men and in 3,974 incidences of prostate cancers (79). It has been reported that proanthocyanidin administration significantly inhibits cell proliferation and induces apoptosis in oral squamous cell carcinoma, skin cancer, and colon cancer cells by attenuating the PI3K pathway and decreasing phosphorylated Protein Kinase B (PKB) ser(473) levels (80, 81). Proanthocyanidins inhibit the growth of prostate cancer cells by reducing the expression of MMP-2 and MMP-9 proteins and regulating androgen receptor-mediated transcription by mediating anti-histone acetyltransferase activity (82). Proanthocyanidins arrest the G0/G1 phase of breast cancer and lung cancer cells by upregulating Cip1/p21 levels and downregulating cyclin levels (78, 83). Additionally, proanthocyanidins effectively suppressed the growth of prostate tumors in male transgenic adenocarcinoma of mouse prostate mice (84). However, further studies are required to elucidate the anticancer mechanisms of proanthocyanidins.

### Anthocyanins

Anthocyanins are members of a complex group of natural phenolic glycosides that are responsible for the black and red color of grapes. Anthocyanins found in grapes are limited in number and consist of mixtures of pigment molecules that vary among grape species and varieties. The most common anthocyanins in red wine are cyanidin, delphinidin, peonidin, malvidin, and petunidin (85). Consumption of wine that is rich in anthocyanins has been associated with a reduced risk of cardiovascular disease and cancer. This can be attributed to the regulation of several signaling pathways and crucial cellular processes, including cell cycle, apoptosis, autophagy, and biochemical metabolism, by anthocyanins (86, 87). The primary pathways targeted by anthocyanins by which it interferes with the growth of cancer cells include mitogen-activated protein kinase (MAPK), nuclear factor κB (NFκB ), AMP-activated protein kinase, and Wnt/β-catenin (88). It has been demonstrated that anthocyanins induce apoptosis of cancer cells by activating caspases and mediating ROS and JNK/p38-MAPK pathway. Moreover, anthocyanins exert metastatic effects through regulating the VEGF signaling pathway and degradation of the extracellular matrix (89). Cyanidin is a common anthocyanidin, which is naturally existed in its glycosylated form as cyanidin-3-glucoside (C3G). C3G is reported to show efficacy in breast cancer, renal cell carcinoma, and colon cancer. C3G inhibits the migratory and invasion of breast cancer cells by inducing mesenchymal to epithelial transition via increasing epithelial-mesenchymal transition (EMT) and Sirt1 expression (90). This glucoside also inhibits proliferation and tumor growth in breast cancer via caspase-3 cleavage and DNA fragmentation (91). A study shows that C3G could be potentially used for the prevention or therapy of colon cancer. This study also reveals that C3G exerts its effect by binding to talin and promoting the interaction of talin with β1A-integrin, which negatively correlates to the survival rate of patients with colon cancer (92). At concentrations of 25–100 μM, Cyanidin-3-O-glucoside (C3G) inhibits the proliferation of renal cell carcinoma cells and tumorigenesis by arresting the cell cycle, inducing apoptosis and autophagy by regulating the expression of early growth response protein 1 (EGR1), selenoprotein W1 (SEPW1), p62 or sequestosome 1, and autophagy related gene 4 (ATG4) (93). C3G suppresses rheumatoid arthritis by reducing IL-6 and IFN-γ, and increasing IL-10 and Tregs (94). However, few studies reported about the immunomodulatory effects of C3G against cancer progression.

Delphinidin has potent antitumor properties associated with the proliferation, migration, and invasion of cancer cells. At low concentrations, delphinidin suppresses the migratory ability and invasiveness of colorectal cancer cells both *in vitro* and *in vivo* by inhibiting the integrin/FAK axis and upregulating the expression of miR-204-3p (95). Delphinidin prominently inhibits the brain-derived neurotrophic factor (BDNF)-induced increase in cell migration and invasion of SKOV3 ovarian cancer cells by decreasing the expression of MMP-2, MMP-9, and AKT pathways (96) and also inhibits proliferation by participating in the PI3K/AKT and ERK1/2 MAPK signaling cascades (97). In addition, delphinidin induces apoptosis and autophagy by downregulating the expression of caspase-3, caspase-9, and AKT/mammalian target of rapamycin (mTOR) pathway in breast cancer cells (98). Finally, delphinidin has also been shown to induce apoptosis by mediating p53 acetylation and oligomerization in prostate cancer cells (99).

There are a few reports on the anticancer effects of peonidin, malvidin, and petunidin at the molecular level. Peonidin is naturally existed as peonidin 3-glucoside (P3G), its glycosylated form. A study shows that P3G can significantly suppress lung cancer metastasis by attenuating ERK 1/2 and AP-1 and activating the MAPK pathway (100). P3G inhibits the proliferation and tumor growth of lung cancer cells by arresting the G2/M phase via the downregulation of cell cycle-related proteins such as CDK-1, CDK-2, and cyclin B1 (101). Both P3G and C3G showed inhibitory effects on human epidermal growth factor receptor (HER)-positive breast cancer cells *in vitro* and *in vivo*. They also promoted the apoptosis of cancer cells and inactivated phospho-HER2 and phospho-AKT (102). Malvidin, at a dose of 200 mg/mL, inhibited the proliferation of several cancer cell lines, including human gastric adenocarcinoma cell line, human colon cancer cell line HCT-116 Michigan cancer foundation - 7 (MCF7) (breast), NCI H460 (lung), and SF-268 (central nervous system) (103). Malvidin-3-galactoside (M3G) inhibited the proliferation, migration, and invasion of HepG2 cells and promoted apoptosis by regulating related proteins, including cleaved caspase-3, MMP-2 and MMP-9, and p-AKT. M3G has also been effective *in vivo* in inhibiting the growth of liver tumors (104). Petunidin-3-O-glucoside (P3OG) could exhibit a significant antiproliferative effect in glioblastoma multiforme (GBM) by regulating glycolytic metabolism. Moreover, P3OG combined with a PI3K inhibitor could significantly induce GBM cell death by regulating the silent information regulator 3 (SIRT3)/p53 and PI3K/AKT/ERK pathways (105). It is well known that the occurrence of cancer induced by persistent oxidative stress is closely related to the inflammatory status. Phenolic compounds as dietary antioxidants exert a pivotal effect in cancer by preventing oxidative stress through scavenging free radicals (106).

Many studies have identified the molecular targets of these compounds and the effects of these compounds on the prevention and treatment of cancer. However, the understanding of molecular mechanisms in this field is still at an embryonic stage, and more studies are needed to better comprehend the anticancer properties of anthocyanins.

## The anticancer effects of non-flavonoids and their potential mechanisms

Diverse non-flavonoids, which have a simpler structure compared to that of flavonoids, have been identified in wine. Derivatives of hydroxycinnamic and hydroxybenzoic acids are the main non-flavonoids present in wine that has not been aged in oak barrels. They are usually present in the vacuoles of grape cells and can be easily extracted when crushed. These compounds play an important role in the oxidation and subsequent browning of grape juice. Hydrolyzable tannins, such as ellagitannin and gallotannin, are present during the aging of wine in oak barrels. Stilbene in wine is produced in grape skin and leaves in response to the infections on grapevines caused by *Botrytis cinerea* and other fungi (2, 107).

### Hydroxycinnamic acids and hydroxybenzoic acid

The most common hydroxycinnamic and hydroxybenzoic acids in wine are caffeic, ferulic, chlorogenic, gallic, vanillic, and coumalic acids. Numerous studies have demonstrated the antitumor efficacy of phenolic acids in breast, ovarian, lung, and oral cancers, and in melanomas. Both caffeic and ferulic acid inhibit the proliferation of melanoma cells by downregulating the CK2-induced phosphorylation of tyrosinase, which is important in melanin biosynthesis (108). Caffeic acid prevents the progression of breast cancer cells and promotes cell death by arresting the cell cycle and reducing cyclin D1, IGFIR, and p-AKT levels (109). Moreover, caffeic acid is capable of disrupting energy homeostasis and regulating oxidative metabolism and glycolysis in cervical tumor cells by activating the AMPK signaling pathway and suppressing the expression of hypoxia inducible factor-1α (HIF-1α), glucose transporter type 1 (GLUT1), hexokinase 2 (HK2), protein kinase M (PKM), and lactate dehydrogenase (LDH) (110). Ferulic acid is a potential candidate for the treatment of several diseases, such as Alzheimer’s disease, cardiovascular diseases, diabetes mellitus, and cancers of the colon and breast. Ferulic acid suppresses the metastasis of breast cancer cells by mediating epithelial to mesenchymal transition (111). It also suppresses cell proliferation and induces apoptosis in osteosarcoma cells by suppressing the PI3K/AKT pathway and downregulating the cell cycle-related proteins, CDK2 and BCL-2 (112). Moreover, it inhibits the proliferation of human cervical carcinoma cells by inducing cell cycle and autophagy. The cell cycle-related proteins, such as cyclin D1, and the autophagy-related proteins, including LC3II and autophagy associated genes (ATG) families, are reported to be regulated by ferulic acid (113). In an *in vivo* study, ferulic acid was administered to male F344 rats with azoxymethane (AOM)-induced colon carcinogenesis, which resulted in the inhibition of tumor growth (114).

Besides caffeic and ferulic acids, several other phenolic acids in wine show potential anticancer effects and the mechanism of anticancer including the induction of cell cycle arrest, inhibition of cell proliferation, reduction of ROS production, induction of apoptosis and autophagy, and the reduction of migration and invasion. The primary mechanisms of chlorogenic acid against cancer include inhibiting the AMPK, hypoxia inducible factor (HIF), VEGF, PI3K, and MAPK/ERK pathways (115). For instance, chlorogenic acid motivates apoptosis in human renal cell carcinoma by activating the caspase protein and inhibiting the PI3K/AKT/mTOR pathway (116). Gallic acid inhibits cancer cell growth by mediating the modulation of genes that encode for cell cycle proteins, metastasis, angiogenesis, and apoptosis. The main mechanism of gallic acid against cancer is by activating the NF-κB and AKT pathways and attenuating the activity of cyclooxygenase, ribonucleotide reductase, and glutathione (117). Gallic acid inhibited the migration and invasion of human nasopharyngeal carcinoma cells by decreasing the expression of MMP-1, AP-1, and E26-AMV virus oncogene cellular homolog (ETS-1) (118). In addition, vanillic acid significantly arrested the G1 phase and inhibited the proliferation of human colon cancer *in vitro* and *in vivo* by suppressing the HIF-1α expression and inhibiting the mTOR/p70S6K/eIF4E binding protein 1 (4E-BP1) and Raf/MEK (Mitogen-activated protein kinase)/ERK pathways (119). Coumaric acid showed its inhibitory effect in the lung (A549), colon (Caco-2), breast (MCF7), hepatic (HepG2), and neuroblastoma (N2a) cancer cell lines. Coumaric acid exerts its anticancer effect as an antioxidant, by depletion of ROS, which has a distinct impact on cellular functions (120).

### Hydrolyzable tannins

Hydrolyzable tannins in wine are only present in wine fermented in oak barrels and not in grapes. They hydrolyze into their respective acid and alcohol components during the aging of wine, which is of great importance for flavor development. Ellagitannins (ET) and gallotannins (GT) are the most common hydrolyzable tannins in wine, which can be hydrolyzed into ellagic and gallic acids, respectively. Many studies show that ET, GT, and their derivatives, such as ellagic and gallic acids, have chemopreventive and chemotherapeutic activities in prostate, colon, breast, oral, esophageal, gastric, liver, cervical, lung, and skin cancers (121–124). ET inhibited the proinflammatory pathways by suppressing the NF-κB. The anti-inflammatory activity could enhance the antioxidant capacity of ET by reducing the levels of free radicals (125). The antiproliferative activity of ET is owing to other mechanisms, which involve its participation in cell cycle arrest, apoptosis, mitochondrial pathways, migration and invasion, metastasis, and angiogenesis. For instance, ET inhibited the proliferation of colon cancer cells and promoted the apoptosis of colon cancer cells by anti-inflammatory effect through activating the AKT pathway and suppressing the NF-κB pathway (126). Another study reports that ET and its derivatives inhibit the proliferation of HT-29 cells by arresting G0/G1 and G2/M phases and regulating the cell cycle-related proteins (127).

Similar to ellagitannins, gallotannins exert anticancer effects by virtue of multiple mechanisms, including the suppression of proliferation and colony formation, arresting cell cycle, inducing apoptosis and autophagy, increasing p21 level and SA-β-Gal activity, and regulating the AIRT1/AMPK pathway (128). However, the health benefits of gallotannins have rarely been discussed because they are not absorbable after consumption (129).

### Stilbenes (Resveratrol)

Resveratrol is the most biologically active stilbene monomer in wine, which is generated when grapevines are infected by *Botrytis* spp. and other fungi (2). All cis and trans isomers of resveratrol have been reported to be found in wine. The average concentration of total resveratrol is 7 mg/L in red wine, 2 mg/L in rose wine, and 0.5 mg/L in white wine (130, 131). The first study in 1997 demonstrated the anticancer activity of resveratrol (11). Since then, for decades, numerous studies have reported the potent and protective effects of resveratrol against various types of cancers. Resveratrol has been shown to be effective in lung, breast, skin, gastric, colon, cervical, uterine, liver, eye, blood, kidney, prostate, brain, bladder, thyroid, head, esophageal, ovarian, and bone cancers (132). Resveratrol exhibits anticancer effects by modulating multiple cell signaling molecules, proteins involved in cell survival and cell proliferation, and various cell signaling pathways. Resveratrol inhibits cancer in the initiation stage by suppressing the oxidative stress by increasing the activity of the antioxidant enzymes and preventing DNA damage by scavenging ROS (133). Resveratrol also decreases the nicotinamide adenine dinucleotide phosphate (NADPH) activity and actuates the Nrf2 signaling pathway (134). Studies show that resveratrol exerts antiproliferative effects at the tumor-promotion stage by arresting the cell cycle via regulating the cell cycle-related proteins and p53-dependent pathway. It also induces apoptosis by activating the mitochondrial apoptosome and the death receptor pathways (135, 136). Resveratrol triggers apoptosis by inhibiting the PI3K/AKT/mTOR pathway (137) and NF-κB activation (138), and activating the MAPK pathway (139). Resveratrol has also been found to modulate the activity of FOXO3a in human breast cancer cells (140). It shows an antiproliferative effect in lung cancer by suppressing the expression of phosphorylation of Rb protein and transcription factors, including AP-1 and NF-κB (141), arresting the G1 phase of the cell cycle through mediating the transforming growth factor-β pathway (142), and inducing the apoptosis by increasing the activities of caspase-3 and the p21/WAF1/CDK-interacting protein (CIP) pathway (143). Resveratrol inhibits cell migration and invasion at the tumor-progression stage by suppressing the extracellular matrix and basement membrane by regulating matrix metalloproteinases (144).

In addition, resveratrol has been reported to induce immune response in tumor cells by reducing the Th17 production and IL-17 secretion, which play a crucial role in immunologic processes (145). It is known that IL-17 can promote tumor angiogenesis by increasing VEGF levels in tumor cells. Studies also show that resveratrol inhibits angiogenesis-related proteins such as VEGF, epidermal growth factor receptor and fibroblast growth factor 2 (FGF-2) (146). IL-17 can enhance the levels of IL-6 and IL-8, while Th17 has been demonstrated to inhibit the antitumor effect by decreasing T-CD8^+^ cells. Th17 also influences the expression of transforming growth factor β (TGF-β), CD39, and CD73 ectonucleotides (147, 148). In tumors, resveratrol has been shown to reduce the TGF-β level *in vivo* and increase the IFN-γ production in T-CD8^+^ cells (149, 150). Resveratrol enhances the function of T-cell by directly targeting programmed cell death 1 ligand 1 (PD-L1) as an immunomodulating mechanism in cancer cells (151).

However, clinical studies on the tumor-inhibition effect of resveratrol in cancer patients are limited. Some studies suggest that patients with colon and prostate cancers treated with resveratrol prior to surgery are benefitted more than those not receiving resveratrol (152). The sample sizes in these clinical trials are not large or representative, therefore, additional studies are warranted.

## Conclusion

**Fig. 3:**
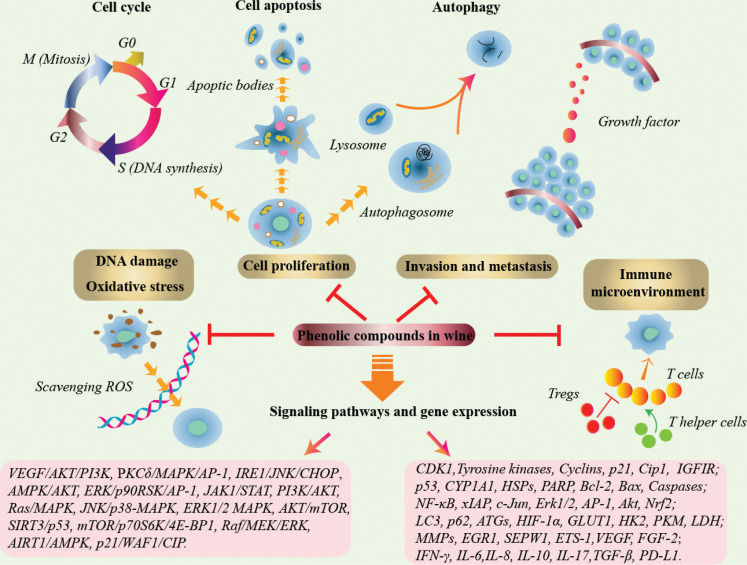
The summary of major mechanisms by phenolic compounds in wine against cancer action.

Wine is the alcoholic drink rich in phenolic compounds. Several studies have suggested that the moderate consumption of wine may have health benefits owing to its phenolic compounds. These phytochemicals showed significant chemoprotective and chemotherapeutic effects in almost all types of human cancers. The major functions of wine phenolic compounds on different cancer types are summarized in Table 2. The mechanisms of action of these phenolic compounds against cancer *in vitro* and *in vivo*, including DNA damage and scavenging ROS, induction of cell proliferation, arresting cell cycle, programmed cell death, invasion and metastasis, immunity and metabolism, regulation of multiple signaling molecules, and gene expression have been discussed (Fig. 3). However, most of these mechanisms are complex, and the effects and underlying molecular mechanisms of some of the phenolic compounds in wine, such as phenolic acids, still remain to be investigated. In the future, the effects of wine on human health, including its anticancer effects, need to be further explored.

**Table 2:** Effects of the main phenolic compounds of wine in different cancer types

Phenolic compounds	Cancer type	Biological effects	Reference
Quercetin	Esophageal squamous carcinoma cells	Proapoptotic effects through mitochondria-mediated pathways or by increasing cytotoxicity and oxidative stress	(40)
	Hepatocarcinoma cells	Proapoptotic effects by increasing the cytotoxicity and oxidative stress, and inhibits cell proliferation by altering the CYP1A1 level	(41, 42)
	Breast cancer cells	Inhibits proliferation by arresting the cell cycle in G1 phase	(43)
	Lung carcinoma cells	Proapoptotic effects by increasing G2/M phase, p53 and p21 levels, and induces cytotoxicity	(44)
	Oral squamous cell carcinoma	Proapoptotic effects by reducing the NF-κB and xIAP levels	(45)
	Prostate cancer	Inhibits the growth, migration, and invasion through the VEGF/Akt/PI3K pathway	(47)
Kaempferol	Breast cancer cells	Arrests the cell cycle, inhibits migration and invasion through the PKCδ/MAPK/AP-1 pathway and the MMP-9 level, and induces apoptosis by regulating the cleavage of PARP, BCL-2, and Bax	(55–57)
	Gastric, hepatic, and lung cancers	Induces autophagy by increasing the conversion of LC3-I to LC3-II	(59–61)
Myricetin	Breast cancer	Suppresses cancer metastasis by downregulating MMP-2/9 and mRNA ST6GALNAC5 levels	(63)
	Ovarian, skin, and colon cancer cells	Induces apoptosis by upregulating the Bax, BCL-2, cleaved PARP, and caspase-3 levels	(54)
Catechin	Lung cancer	Induces apoptosis by reducing the levels of c-Jun and Erk1/2	(69)
	Colon cancer	Induces apoptosis by suppressing phospho-Akt and nuclear β-catenin levels	(70)
	Prostate cancer	Inhibits the IGF/IGF1R axis and restrains the VEGF-dependent angiogenesis	(71)
Epicatechin	Hepatocarcinoma cells	Inhibits proliferation by regulating the NF-κB, AP-1, Akt, and Nrf2 pathways	(74)
Proanthocyanidins	Oral squamous cell carcinoma, skin cancer, and colon cancer cells	Inhibits proliferation and induces apoptosis by regulating the PI3K pathway and decreasing PKB ser (473) phosphorylation levels	(80, 81)
	Prostate cancer cells	Reduces the MMP-2 and MMP-9 levels	(82)
	Breast cancer and lung cancer cells	Arrests the G0/G1 phase by increasing Cip1/p21 levels and decreasing cyclin levels	(78, 83)
Cyanidin-3-glucoside	Breast cancer cells	Inhibits migration and invasion by increasing the EMT and Sirt1 expression, and inhibits proliferation via caspase-3 cleavage and DNA fragmentation	(90, 91)
	Renal cell carcinoma cells	Inhibits proliferation by regulating the EGR1, SEPW1, p62, and ATG4 levels	(93)
Delphinidin	Colorectal cancer	Inhibits the integrin/FAk axis and upregulates the expression of miR-204-3p	(95)
	Ovarian cancer cells	Decreases the expression of MMP-2, MMP-9, AKT pathways, PI3K/AKT, and ERK1/2 MAPK pathways	(96, 97)
	Breast cancer cells	Induces apoptosis and autophagy	(98)
	Prostate cancer cells	Mediates p53 acetylation and oligomerization	(99)
Peonidin 3-glucoside	Lung cancer	Regulates ERK 1/2 and AP-1 levels, the MAPK pathway, and cell cycle-related proteins	(99, 101)
Malvidin 3-galactoside	Hepatocellular carcinoma	Regulates the cleaved caspase-3, MMP-2 and MMP-9, and p-AKT levels	(104)
Petunidin-3-*O*-glucoside	Glioblastoma multiforme	Inhibits proliferation by regulating glycolytic metabolism	(105)
Caffeic acid	Breast cancer cells	Reduces cyclin D1, IGFIR, and p-AKT levels	(109)
	Cervical tumor cells	Activates the AMPK pathway and the expression of HIF-1α, GLUT1, HK2, PKM, and LDH	(110)
Ferulic acid	Osteosarcoma cells	Suppresses the PI3K/AKT pathway and downregulates the CDK2 and BCL-2 levels	(112)
	Cervical carcinoma cells	Induces cell cycle and autophagy	(113)
Ellagitannins	Colon cancer cells	Inhibits the proinflammatory pathways, activates the AKT pathway, and suppresses the NF-κB pathway	(125, 126)
Gallotannins	Hepatocellular carcinoma cells	Increases p21 level and SA-β-Gal activity and regulates the AIRT1/AMPK pathway	(128)
Resveratrol	Lung cancer cells	Decreases the NADPH activity and actuates the Nrf2 pathway	(134)
	Breast cancer cells	Modulates the activity of FOXO3a and inhibits the expression of VEGF, EGFR, and FGF-2	(140, 146)
	Epidermoid carcinoma	Increases the caspase-3 level and the p21/WAF1/CIP pathway	(143)
	Colorectal cancer	Increases the levels of IL-6, IL-8, and Th17	(147)

Despite the beneficial anticancer effects of phenolic compounds in wine, many challenges have yet to be overcome. The first challenge is bioavailability, and there are still limited studies on their pharmacokinetics in humans. The absorption and bioavailability of wine-derived phenolic compounds are not well but better with daily diet. Nanoparticles have been extensively studied to deliver anticancer drugs because of their enhanced anticancer potential and promising clinical application. The other challenge is the use of phenolic compounds as an adjuvant in radiotherapy or chemotherapy with established anticancer drugs such as cisplatin and gefitinib. The combined therapies have great prospects and potential because the use of phenolic compounds can reduce the dose of anticancer drugs and, therefore, reduce side effects. In addition, experimental and clinical studies to further study the anticancer potential of wine-derived phenolic compounds and elucidate their mechanisms of action are required.
